# Ethyl acetate extract from *Platycladus orientalis* leaves ameliorates diabetic cardiomyopathy via alleviating oxidative stress and suppressing myocardial fibrosis

**DOI:** 10.3389/fphar.2026.1771512

**Published:** 2026-05-13

**Authors:** Chao Wang, Yi-Xuan Sun, Jia-Hui Guo, Xin-Wei Shen, Li Li

**Affiliations:** 1 Department of CT, Rizhao People’s Hospital, Rizhao, Shandong, China; 2 College of Pharmacy, Jining Medical University, Rizhao, China

**Keywords:** diabetes cardiomyopathy, myocardial fibrosis, Nrf2/HO-1 signaling pathway, oxidative stress, platycladus orientalis leaves

## Abstract

**Introduction:**

Diabetic cardiomyopathy (DCM) remains a serious clinical challenge with limited effective therapeutic options. This study investigated the cardioprotective effects and underlying mechanisms of ethyl acetate extract from *Platycladus orientalis* leaves (EAEPOL) in a murine DCM model.

**Methods:**

C57BL/6 mice were randomized into control, DCM, and EAEPOL treatment groups. The chemical constituents of EAEPOL were identified by liquid chromatography-tandem mass spectrometry (LC-MS/MS). Random blood glucose levels were monitored longitudinally throughout the intervention. Cardiac function, myocardial fibrosis, oxidative stress, endothelial integrity markers and fibrosis-related markers were assessed comprehensively. The Nuclear factor erythroid 2-related factor 2 (Nrf2)/heme oxygenase-1 (HO-1) pathway was analyzed by Western blot and quantitative Reverse Transcription polymerase chain reaction (qRT-PCR).

**Results:**

LC-MS/MS identified 60 compounds in EAEPOL, with ginkgetin, cyanin, myricitrin, and amentoflavone exhibiting relatively high contents. Compared with the control group, DCM mice exhibited marked and persistent hyperglycemia, significant cardiac dysfunction, myocardial fibrosis, and oxidative stress and disrupted endothelial/fibrotic marker expression (*P* < 0.05, *P* < 0.01, *P* < 0.001 or *P* < 0.0001). Compared with the DCM group, EAEPOL treatment significantly lowered random blood glucose levels in a time-dependent manner, improved cardiac function, reduced fibrosis, enhanced antioxidant capacity, modulated Nrf2/HO-1 signaling, and restored endothelial/fibrotic marker expression (*P* < 0.05, *P* < 0.01, *P* < 0.001 or *P* < 0.0001).

**Conclusion:**

EAEPOL exerts significant protective effects against DCM via reducing hyperglycemia, Nrf2/HO-1-mediated antioxidant responses and suppression of myocardial fibrotic remodeling. These findings suggest that EAEPOL represents a potential natural cardioprotective agent for DCM that warrants further preclinical investigation.

## 1 Introduction

Diabetes has evolved into a major global public health challenge, exerting a substantial burden on human health. Recent 2020 epidemiological data indicate that the prevalence of diabetes among Chinese adults has reached 12.8%, placing China at the forefront of this worldwide epidemic. Diabetic cardiomyopathy (DCM), a severe and independent cardiovascular complication of diabetes, is characterized by diffuse myocardial lesions including diffuse interstitial fibrosis, cardiomyocyte hypertrophy, microvascular dysfunction, and impaired cardiac contractility. It constitutes a core pathological driver that ultimately progresses to heart failure and increased mortality in diabetic patients. Indeed, cardiovascular complications, particularly DCM, account for nearly 80% of diabetes-related deaths (Zhang et al., 2024). Accordingly, the development of mechanism-based, targeted therapeutic strategies for DCM constitutes an urgent and unmet clinical need.

Despite growing recognition of DCM as a distinct pathological entity, the clinical management of this prevalent cardiovascular complication remains a major unmet challenge. A recent systematic review demonstrated that over 40% of patients with type 2 diabetes present with diastolic dysfunction in the absence of impaired cardiac systolic function, and early-stage DCM is often asymptomatic yet can progress insidiously to symptomatic heart failure with a 5-year mortality rate comparable to or even exceeding that of many common malignancies (Chen et al., 2025). Critically, no specific therapeutic regimens for DCM currently exist beyond conventional heart failure management (Chen et al., 2025). Current therapeutic strategies primarily manage DCM as a secondary complication of diabetes, incorporating glucose-lowering drugs such as sodium-glucose cotransporter 2 inhibitors (SGLT2i) and glucagon-like peptide-1 receptor agonists (GLP-1 RA), in conjunction with standard heart failure therapies including angiotensin-converting enzyme inhibitors (ACEi), angiotensin II receptor blockers (ARBs), and beta-blockers (Smati et al., 2025). However, these approaches often fail to fully reverse the underlying structural and molecular abnormalities of DCM, and many are limited by side effects or incomplete targeting of core pathogenic pathways (Balakrishnan et al., 2025; Abdullah et al., 2025). Collectively, these limitations underscore the urgent need for safer, more effective, and pathophysiologically targeted interventions to prevent and treat DCM.

Accumulating evidence identifies hyperglycemia-induced oxidative stress as a central mechanistic driver of both contractile impairment and progressive myocardial fibrosis in DCM (Song et al., 2024; Puhari et al., 2023). Sustained oxidative stress leads to excessive reactive oxygen species (ROS) accumulation, which triggers cellular injury via multiple mechanisms including ATP depletion, DNA damage, mitochondrial dysfunction, and the promotion of necrotic and apoptotic cell death (Morales et al., 2014). Additionally, excessive ROS also activates pro-fibrotic signaling cascades, notably transforming growth factor-β (TGF-β) signaling, which stimulates the differentiation of cardiac fibroblasts into myofibroblasts and promotes excessive extracellular matrix deposition in the myocardium (Liu et al., 2022). Notably, oxidative stress and interstitial fibrosis are shared pathological drivers across multiple forms of myocardial injury. These processes are closely intertwined with signaling pathways such as TGF-β and epithelial-mesenchymal transition (EMT), which have been widely implicated in both cardiac remodeling and tumor cell invasion and metastasis (Peng et al., 2022; Frangogiannis, 2022).

*Platycladus orientalis* leaf is a traditional Chinese medicinal herb renowned for its diverse pharmacological properties, including antioxidant, anti-inflammatory, hypoglycemic, and tissue-protective activities (Zhang et al., 2021). A comprehensive systematic review has confirmed that flavonoids, diterpenes, and volatile oils represent the primary bioactive constituents responsible for these effects (Shan et al., 2014a). Consistent with this, our previous study demonstrated that aqueous extracts of *Platycladus orientalis* leaves exert hypoglycemic and hypolipidemic effects *in vivo* (Li et al., 2017). Wang et al. (Wang, 2012) screened different solvent extracts *in vitro* and found that the ethyl acetate fraction exhibited the strongest α-glucosidase inhibitory activity. Similarly, Gu et al. (Gu and Sun, 2015) reported that the same ethyl acetate fraction displayed superior antioxidant capacity compared with other polar fractions. Recent studies have further confirmed that the ethyl acetate fraction of *Platycladus orientalis* leaves exerts relatively high antioxidant, anti-diabetic, anti-inflammatory, and anti-glycation activity, with these bioactivities showing a significant positive correlation with flavonoid content (Ho et al., 2022). Our preliminary liquid chromatography-tandem mass spectrometry (LC-MS/MS) analysis of the ethyl acetate extract from *Platycladus orientalis* leaves (EAEPOL) confirmed a rich repertoire of flavonoid compounds, including ginkgetin, myricitrin, amentoflavone and cyanin, as well as notable antioxidant secondary metabolites such as ligustroside and ganoderic acid H. Based on these preclinical findings, we hypothesized that EAEPOL may protect against DCM by lowering blood glucose, attenuating oxidative stress and inhibiting pathological myocardial fibrosis.

Cardiac microvascular endothelial integrity is essential for maintaining normal myocardial structure and function. In diabetes, endothelial damage is an early event that promotes myocardial inflammation, ROS accumulation, and subsequent fibrotic remodeling. Endothelial markers including CD31 and CDH5 (VE-cadherin) are critical indicators of endothelial junction stability and vascular homeostasis, while α-SMA and FSP1 are widely used to assess myofibroblast activation and extracellular matrix production (Xu and Kovacic, 2023; Alvandi and Bischoff, 2021; Giordo et al., 2021). Modulation of these markers reflects the degree of myocardial structural protection and anti-fibrotic efficacy, which can be used to evaluate therapeutic responses in DCM.

Cardio-oncology is an emerging interdisciplinary field focused on the prevention, diagnosis, and management of cardiovascular toxicity induced by cancer therapies (Quagliariello et al., 2025). Published evidence has established that oxidative stress, endothelial dysfunction, and myocardial fibrosis are core pathological events in both DCM and cancer therapy-related cardiotoxicity (L'Abbate et al., 2020; Singh et al., 2023). These same processes are also closely linked to tumor cell invasion and metastasis through interconnected signaling networks (Peng et al., 2022; Frangogiannis, 2022). Oxidative stress and progressive fibrosis are well-recognized mediators of anthracycline-induced cardiomyopathy, paralleling key mechanisms underlying DCM (Hara, 2025). Endothelial impairment further contributes to cardiac functional decline in both disease contexts (Slavcheva and Angelov, 2023).

Although *Platycladus orientalis* leaves have been widely studied for their antioxidant and anti-fibrotic properties, the potential cardioprotective effects of EAEPOL in diabetic cardiomyopathy remain poorly defined. In particular, whether EAEPOL can alleviate DCM by suppressing oxidative stress, preserving endothelial integrity, and reducing myocardial fibrosis has not been systematically investigated. Accordingly, the present study was designed to evaluate the cardioprotective effects of EAEPOL in a murine model of type 1 DCM and to explore its underlying mechanisms with a focus on oxidative stress pathways and myocardial fibrotic remodeling. This study characterizes the anti-oxidative and anti-fibrotic effects of EAEPOL in DCM, while aligning with cardio-oncology-related literature that emphasizes shared mechanisms of myocardial injury across metabolic and cancer therapy-associated cardiac conditions. Therefore, EAEPOL may provide a mechanistic reference for exploring interventions targeting shared pathological processes in cancer therapy-related cardiac injury, as well as in conditions linked to tumor cell invasion and metastasis.

## 2 Methods

### 2.1 Animal procedure and drug treatment

Thirty healthy adult C57BL/6 mice (mean body weight ≈20 g, both sexes) were obtained from Jinan Pengyue Experimental Animal Breeding Co., Ltd. (Jinan, China; License No. SYXK(Lu)-2018-0002). The mice were housed under specific pathogen-free (SPF) conditions with a controlled temperature of 22 °C ± 1 °C, relative humidity of 55% ± 5%, and a 12-h light/dark cycle, with *ad libitum* access to a standard laboratory rodent chow and autoclaved drinking water. Following a minimum 7-day acclimation period, all experimental protocols were performed in strict compliance with the National Institutes of Health Guide for the Care and Use of Laboratory Animals and were approved by the Animal Ethics Committee of Jining Medical University (Approval No. JNMC-2024-DW-253). All experimental procedures were designed to minimize animal suffering.

Fresh leaves of *Platycladus orientalis* (collected from the Chinese Medicinal Herb Garden, Jining Medical University) were coarsely chopped and subjected to triple sequential extraction with boiling distilled water (1.5 h per extraction). The combined aqueous extracts were filtered to remove residual plant debris. The filtrate was concentrated under reduced pressure using a rotary evaporator at 40 °C. The concentrate was then partitioned three times with an equal volume of petroleum ether in a separatory funnel; the lower aqueous phase was retained and pooled after each partitioning step. Subsequently, the pooled aqueous phase was extracted three times with an equal volume of ethyl acetate (1:1, v/v), and the upper ethyl acetate layer was collected and combined after each extraction. The combined ethyl acetate fractions were concentrated to a syrupy consistency using rotary evaporation (40 °C) and finally lyophilized to a dry powder. The resulting EAEPOL was stored in airtight containers at 4 °C for subsequent experimental use.

The purity of EAEPOL was assessed by thin-layer chromatography (TLC) in accordance with the *Chinese Pharmacopoeia* (2020 edition, Volume I), and the semi-quantitative analysis of major bioactive components was performed via UPLC-QTOF-MS/MS (as described in Section 2.2) to ensure the consistency of the extract. The specific analytical criteria were as follows: silica gel G thin-layer plates were used with a developing solvent system of chloroform-methanol-water (13:6:2, v/v/v) and 1% aluminum nitrate ethanol solution as the chromogenic reagent. After developing and air-drying, the EAEPOL sample showed no distinct impurity bands under ultraviolet light at 254 nm, confirming that the extract purity met the experimental requirements. The extraction yield of EAEPOL was calculated using the following formula: *Extraction yield (%)* = *(Dry weight of purified EAEPOL*/*Dry weight of raw Platycladus orientalis leaf material) × 100%*. Under the optimized conditions, the average extraction yield of EAEPOL was 6.2% ± 0.3% (n = 3), which confirmed good reproducibility of the extraction protocol.

Eight-week-old SPF C57BL/6 mice were used for the *in vivo* experiments, with both sexes included to eliminate potential gender-associated experimental bias. Mice were randomly assigned to three groups (n = 10 per group): (Zhang et al., 2024): normal control group; (Chen et al., 2025); DCM model group; and (Smati et al., 2025) EAEPOL treatment group. Diabetes was induced by daily intraperitoneal injection of freshly prepared streptozotocin (STZ) (catalog no. S817944-1g, purity ≥98%, Shanghai McLean Biochemical Technology Co., Ltd.) dissolved in 50 mM sodium citrate buffer (pH 4.5) at a dose of 80 mg/kg daily for 5 consecutive days. Mice in the control group received an equivalent volume of citrate buffer alone. Random blood glucose levels were monitored daily using a glucometer (Sinocare Biosensing Co., Ltd.). Successful induction of diabetes was defined as a persistent random blood glucose concentration exceeding 15 mM for two consecutive weeks. Mice meeting this criterion were then re-randomized into the DCM model group (n = 10) and EAEPOL treatment group (n = 10). Notably, the normal control group did not receive EAEPOL intervention to evaluate the basal cardiac status without drug exposure, and the STZ-induced diabetes model was confirmed by persistent hyperglycemia (random blood glucose >15 mM for two consecutive weeks) to ensure model stability. For the subsequent 6-week intervention period, EAEPOL was suspended in 5% sodium carboxymethyl cellulose (CMC-Na) and administered via daily oral gavage at a dose of 375 mg/kg. This dosage was selected based on our previous dose-response and safety assessment studies in diabetic murine models, which identified 375 mg/kg/day as the optimal effective dose with no observable adverse effects (Liu et al., 2024). The normal control and DCM model groups received an equal volume of the 5% CMC-Na vehicle via the same gavage schedule for the same duration. Random blood glucose levels were measured weekly from the tail vein using a glucometer throughout the intervention period.

### 2.2 LC-MS/MS analysis of the EAEPOL

Sample Preparation: An accurately weighed aliquot (0.2 g) of the EAEPOL was transferred to a conical flask, and 20 mL of HPLC-grade methanol was added. The flask was sealed, weighed, and subjected to ultrasonication for 40 min at room temperature. After cooling to room temperature, the flask was reweighed, and the lost solvent was replenished with HPLC-grade methanol to the original weight. The resulting solution was filtered through a 0.22 μm organic filter membrane, and an aliquot of the filtrate was collected for Ultra-Performance Liquid Chromatography Quadrupole Time-of-Flight Tandem Mass Spectrometry (UPLC-QTOF-MS/MS) analysis.

Chromatographic Conditions: Chromatographic separation was performed on a Waters ACQUITY UPLC® H-Class system equipped with a photodiode array (PDA) detector. Analyte separation was achieved using an ACQUITY UPLC® BEH C18 column (2.1 × 100 mm, 1.7 μm particle size) maintained at 30 °C. The mobile phase consisted of (A) 0.1% formic acid in ultrapure water and (B) HPLC-grade methanol, delivered at a constant flow rate of 0.3 mL/min according to the gradient elution program detailed in Table 1. The detection wavelength was set at 258 nm, and the injection volume was 1 μL.

**TABLE 1:** Gradient elution program for LC-MS/MS analysis.

Time (min)	Mobile Phase A (%)	Mobile Phase B (%)
0–2	95–70	5–30
2–7	70–66	30–34
7–15	66–20	34–80

Mass Spectrometric Conditions: Mass spectrometric analysis was conducted on a Waters Xevo G2-XS Quadrupole Time-of-Flight (QTOF) mass spectrometer equipped with an electrospray ionization (ESI) source operating in negative ion mode (ESI^−^). The optimized MS parameters were as follows: capillary voltage, 3.0 kV; cone voltage, 40 V; source temperature, 120 °C; desolvation temperature, 450 °C; desolvation gas flow, 600 L/h; cone gas flow, 50 L/h. Mass spectral data were acquired over a mass-to-charge ratio (m/z) range of 100–1,000 for a total run time of 18 min. Real-time mass calibration was performed using LockSpray™ interface with leucine enkephalin (LE) solution as the reference compound.

### 2.3 Echocardiographic assessment of cardiac structure and function

Cardiac structure and function were assessed following the 6-week treatment period using a high-resolution small-animal ultrasound imaging system (Vevo 2100™, Visual Sonics, Canada). Prior to imaging, mice were anesthetized with 3% isoflurane for induction, followed by maintenance with 1.5% isoflurane in medical oxygen. The chest fur was removed with a depilatory agent to ensure optimal acoustic coupling between the transducer and the chest wall. Two-dimensional guided M-mode and B-mode echocardiography were performed to quantify cardiac structural and functional parameters, as previously described (Bonagura and Visser, 2022). The following parameters were measured: heart rate (HR, beats/min), left ventricular ejection fraction (EF, %), left ventricular anterior wall thickness at systole (LVAWs, mm) and diastole (LVAWd, mm), and left ventricular posterior wall thickness at systole (LVPWs, mm) and diastole (LVPWd, mm). All measurements were performed by an experienced investigator who was blinded to the experimental group assignments to eliminate observer bias. Each parameter was measured in three consecutive cardiac cycles, and the average value was used for statistical analysis to ensure reliability.

### 2.4 Quantification of cardiac oxidative stress markers

Following echocardiography, mice were deeply anesthetized with 5% isoflurane (anaesthetic depth confirmed by absence of pedal reflex) and euthanized by cardiac puncture. Hearts were rapidly excised, rinsed in ice-cold phosphate-buffered saline (PBS, pH 7.4), snap-frozen in liquid nitrogen, and stored at −80 °C until biochemical analysis.

For oxidative stress marker assays, approximately 20 mg of left ventricular myocardial tissue was weighed and homogenized in 200 μL of ice-cold PBS (pH 7.4) using an automatic sample grinding system (Servicebio, Wuhan, China). The homogenate was centrifuged at 12,000 × g for 15 min at 4 °C, and the resulting supernatant was collected for subsequent biochemical measurements.

The activities of superoxide dismutase (SOD) (catalog No. A001-1-1) and total antioxidant capacity (T-AOC) (catalog No. A015-1-2), as well as the level of malondialdehyde (MDA) (catalog No. A003-2), were quantified using commercial assay kits (Jiancheng Bioengineering Institute, Nanjing, China) in strict accordance with the manufacturers’ standardized protocols. All spectrophotometric measurements were performed in duplicate to ensure analytical reproducibility.

### 2.5 Protein expression analysis by western blotting

Total protein was extracted from frozen myocardial tissues by homogenization in ice-cold RIPA lysis buffer (Beyotime Biotechnology, Shanghai, China) supplemented with protease and phosphatase inhibitors. The homogenates were centrifuged at 12,000 × g for 15 min at 4 °C, and the resulting supernatant was collected as the total protein extract. Protein concentration was determined using a bicinchoninic acid (BCA) assay kit (Beyotime Biotechnology) according to the manufacturer’s instructions.

Protein samples (20–30 μg per lane) were separated by 10% sodium dodecyl sulfate-polyacrylamide gel electrophoresis (SDS-PAGE) and electrophoretically transferred onto polyvinylidene difluoride (PVDF) membranes (0.45 μm pore size). Following transfer, the membranes were blocked with 5% (w/v) non-fat dry milk in TBST buffer (10 mM Tris-HCl, 150 mM NaCl, 0.1% Tween-20, pH 7.5) for 1 h at room temperature to block non-specific protein binding.

The blocked membranes were incubated overnight at 4 °C with the following primary antibodies (all purchased from Beyotime Biotechnology) diluted in TBST: rabbit anti- Nuclear factor erythroid 2-related factor 2 (Nrf2, catalog No. AF0636, 1:1000), rabbit anti- heme oxygenase-1 (HO-1, catalog No. AG2181, 1:1000), rabbit anti-CD31 (catalog No. AF1642, 1:1000), rabbit anti-α-SMA (catalog No. AF0048, 1:1000). CD31 was used as a marker of endothelial integrity, and α-SMA was used as a marker of myofibroblast activation and myocardial fibrosis. Rabbit anti-Glyceraldehyde-3-phosphate dehydrogenase (GAPDH, catalog No. AF0153, 1:1000) was used as the loading control for protein normalization.

After three consecutive washes with TBST (10 min each), the membranes were incubated with horseradish peroxidase (HRP)-conjugated goat anti-rabbit IgG secondary antibody (1:1000, Beyotime Biotechnology) for 1 h at room temperature. Following an additional series of TBST washes, immunoreactive bands were visualized using an enhanced chemiluminescence (ECL) detection kit (New Saimei Biotechnology). Band images were captured using an automatic chemiluminescence imaging system (FL1000, Thermo Fisher Scientific). Band intensities were quantified using ImageJ software (v1.53, National Institutes of Health, USA), and all Western blot experiments were performed in three independent biological replicates to ensure reproducibility. Relative protein expression levels were normalized to GAPDH and presented as the ratio of target protein to GAPDH optical density.

### 2.6 Quantitative real-time PCR analysis

Total RNA was isolated from frozen murine myocardial tissues using TRIzol ® reagent (Vazyme Biotech, Nanjing, China) according to the manufacturer’s standard protocol. RNA purity and concentration were determined spectrophotometrically using a NanoDrop 2000 UV-Vis spectrophotometer (Thermo Fisher Scientific, USA); only RNA samples with an A260/A280 absorbance ratio between 1.9 and 2.1 were used, indicating minimal protein or phenolic contamination. First-strand complementary DNA (cDNA) was synthesized from 1 μg of high-quality total RNA using the Hiscript III Reverse Transcriptase System (catalog No. R302-01, Vazyme Biotech, Nanjing, China) following the manufacturer’s recommended thermal cycling conditions.

Quantitative real-time polymerase chain reaction (qRT-PCR) was performed using ChamQ Universal SYBR qPCR Master Mix (catalog No. Q711-02, Vazyme Biotech, Nanjing, China) on a QuantStudio 6 Real-Time PCR system (Applied Biosystems, USA). The thermal cycling protocol was as follows: initial denaturation at 95 °C for 30 s, followed by 40 cycles of denaturation at 95 °C for 10 s and combined annealing/extension at 60 °C for 30 s. A melting curve analysis (95 °C for 15 s, 60 °C for 1 min, 95 °C for 15 s) was performed after each run to confirm the specificity of the PCR products and exclude non-specific amplification. β-Actin was used as the endogenous reference gene for mRNA expression normalization. All primer sequences used in this study are listed in Table 2. Relative mRNA expression levels were calculated using the 2^−ΔΔCt^ method and presented as the fold change relative to the normal control group.

**TABLE 2:** qRT-PCR primer sequences for mouse target genes.

Gene	Forward primer (5′-3′)	Reverse primer (5′-3′)
Actb	GTG​ACG​TTG​ACA​TCC​GTA​AAG​A	GTA​ACA​GTC​CGC​CTA​GAA​GCA​C
Cdh5	CCT​TGG​GCT​TTC​TGA​CTG​TTG​T	CAG​GGA​CTT​CGT​GGG​TTT​GAT
S100a4	GTG​TCC​ACC​TTC​CAC​AAA​TAC​TCA	TGC​AGC​TTC​ATC​TGT​CCT​TTT​C
Hmox1	GCT​AAG​ACC​GCC​TTC​CTG​CT	ACG​AAG​TGA​CGC​CAT​CTG​TGA
Nfe2L2	TGT​CTT​AAT​ACC​GAA​AAC​AAG​CAG​C	GAC​CAC​AGT​TGC​CCA​CTT​CTT​TT

Cdh5(Cadherin-5), S100a4 is also known as fibroblast-specific protein 1 (FSP1); Hmox1 encodes heme oxygenase-1 (HO-1); Nfe2L2 encodes nuclear factor erythroid 2-related factor 2 (Nrf2).

### 2.7 Histopathological, immunohistochemical and immunofluorescence analysis of cardiac tissues

Excised heart tissues were fixed in 4% paraformaldehyde for 24 h at 4 °C, followed by gradient dehydration in ethanol, clearing in xylene, and embedding in paraffin wax. The paraffin-embedded tissue blocks were sectioned into 5-μm thick serial sections using a microtome and mounted onto poly-L-lysine-coated glass slides for subsequent histological and immunohistochemical (IHC) and immunofluorescence (IF) analyses.

Histological staining was performed using Hematoxylin and Eosin (H&E; Phygene, China), Masson’s Trichrome (Solarbio, China), and Picro-Sirius Red (Phygene, China) staining kits, in strict compliance with the manufacturers’ protocols. Masson’s Trichrome and Picro-Sirius Red staining was employed to assess collagen deposition under conventional brightfield microscopy, with collagen fibers staining blue (Masson’s) and red (Picro-Sirius Red), respectively, allowing clear visualization of interstitial collagen deposition.

For semi-quantitative analysis of collagen deposition (Masson’s trichrome staining and Picro-Sirius Red staining), the field selection criteria and analytical procedures were performed as previously described with minor modifications (Sobierajska et al., 2022). Briefly, strict field selection criteria were applied: only intact myocardial tissue sections without technical artifacts (e.g., folding, tearing, or non-specific staining) were included; fields were randomly selected from the left ventricular free wall-the primary site of myocardial fibrosis in DCM-to avoid selection bias, with vascular and perivascular areas excluded to focus on interstitial myocardial fibrosis. For each staining method, three non-overlapping low-power fields (LPF, ×100 magnification) were randomly selected from each myocardial section; ten mice were included per group with three sections per mouse, resulting in a total of 90 fields (10 mice × 3 sections × 3 fields) analyzed per staining method. Collagen-positive areas were quantified using ImageJ software (NIH, USA), and the degree of myocardial fibrosis was expressed as the ratio of collagen-positive area to the total myocardial tissue area in each field. All image analyses were performed by an investigator blinded to the experimental groups to minimize observer bias.

Serial paraffin sections of myocardial tissue were used for both IHC and IF analyses, with the following common experimental protocol: Deparaffinized and rehydrated sections underwent antigen retrieval by microwave heating in citrate-based buffer (pH 6.0) for 15 min. After cooling to room temperature, tissue sections were encircled with a hydrophobic barrier using a PAP pen (Servicebio, China) and incubated with 5% bovine serum albumin (BSA) in PBS for 1 h at room temperature to block non-specific binding. Sections were then incubated with the appropriate primary antibody overnight at 4 °C.

For IHC, following primary antibody incubation with rabbit anti-Collagen I (catalog No. GB11022-3, 1:100; Servicebio, China) and rabbit anti-Collagen III (catalog No. GB111629, 1:100; Servicebio, China), endogenous peroxidase activity was quenched with 3% hydrogen peroxide (H_2_O_2_) for 15 min at room temperature. Sections were then incubated with corresponding HRP-conjugated secondary antibodies for 2.5 h at room temperature. Immunoreactive signals were visualized using 3,3′-diaminobenzidine (DAB) chromogen substrate, with the development of a brown-yellow precipitate indicating a positive immunoreaction. The chromogenic reaction was terminated by rinsing with distilled water, and sections were subsequently counterstained with hematoxylin, differentiated in 1% acid alcohol, blued in ammonia water, gradient dehydrated, cleared in xylene, and mounted with neutral balsam for light microscopic examination. Collagen I and Collagen III expression was semi-quantified by measuring the integrated optical density (IOD) of positive staining using ImageJ software, normalized to the total tissue area in each field.

For IF, following the 5% BSA blocking step, sections were incubated with rabbit anti-α-SMA primary antibody (catalog No. AF0048, 1:1000; Beyotime, China) overnight at 4 °C. α-SMA was used as a specific marker of myofibroblast activation, a key driver of myocardial fibrosis. After three washes with PBS (10 min each), sections were incubated with appropriate fluorophore-conjugated secondary antibodies for 50 min at room temperature in the dark to prevent fluorophore photobleaching. Cell nuclei were counterstained with 4′,6-diamidino-2-phenylindole (DAPI) for 10 min at room temperature. Finally, sections were mounted with anti-fade mounting medium and visualized under a fluorescence microscope (Olympus, Japan). Digital images were acquired and analyzed using ImageJ software (NIH, USA). In the resulting micrographs, DAPI-stained nuclei exhibited blue fluorescence, while α-SMA positive signal was visualized as red fluorescence. The percentage of α-SMA-positive area relative to the total myocardial tissue area was calculated to quantify myofibroblast activation.

### 2.8 Myocardial hydroxyproline content determination

Myocardial hydroxyproline content-a specific biochemical marker of collagen synthesis-was quantified using a commercial hydroxyproline assay kit (catalog No. A030-1, Nanjing Jiancheng Bioengineering Institute, China) according to the manufacturer’s protocol with minor modifications based on previous high-impact studies (Conrad et al., 1995).

Briefly, following the 6-week intervention period, mice were anesthetized with 1% pentobarbital sodium (50 mg/kg, i. p.). Approximately 50 mg of left ventricular myocardium was isolated, rinsed with ice-cold saline, blotted dry with filter paper, and homogenized in ice-cold distilled water. The homogenate was hydrolyzed with an equal volume of 6 M HCl at 100 °C for 24 h. After cooling to room temperature and centrifugation at 3,000 × g for 10 min, the clear supernatant was collected for subsequent analysis.

Hydroxyproline in the hydrolyzate was derivatized and color-developed using chloramine-T and p-dimethylaminobenzaldehyde (DMAB) reagents according to the kit protocol. The absorbance was measured at 550 nm using a microplate reader (Bio-Rad, USA). A standard curve was generated using serial dilutions of a pure hydroxyproline standard, and myocardial hydroxyproline content was calculated from the standard curve and expressed as mg hydroxyproline per gram of wet myocardial tissue. All samples were assayed in triplicate with appropriate blank and standard controls included in each assay to ensure analytical accuracy and reliability.

### 2.9 Statistical analysis

All statistical analyses were performed using GraphPad Prism software (version 8.0.2, GraphPad Software, USA). Continuous variables are presented as mean ± standard deviation. Prior to parametric statistical testing, the underlying assumptions of normality and homogeneity of variances were verified: data normality was assessed using the Shapiro-Wilk test, and homogeneity of variances was evaluated using Levene’s test (all P > 0.05, confirming compliance with parametric test assumptions). Intergroup comparisons of continuous variables were performed using one-way analysis of variance (ANOVA). When a significant main effect was detected (P < 0.05), Tukey’s honestly significant difference (HSD) *post hoc* test was used for pairwise multiple comparisons to correct for multiple testing bias, ensuring the reliability of intergroup comparisons. A two-tailed P < 0.05 was considered statistically significant for all analyses.

## 3 Results

### 3.1 Chemical composition analysis of EAEPOL by LC-MS/MS

**FIGURE 1:**
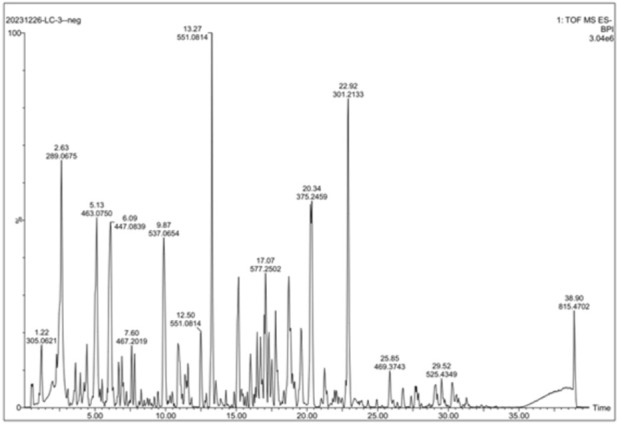
Representative base peak intensity (BPI) total ion chromatogram (TIC) of EAEPOL analyzed by UPLC-QTOF-MS in negative electrospray ionization (ESI^−^) mode. Chromatographic separation was performed on an ACQUITY UPLC BEH C18 column (2.1 × 100 mm, 1.7 μm) using a gradient elution of 0.1% formic acid in ultrapure water **(A)** and methanol **(B)** at a flow rate of 0.3 mL/min (gradient program detailed in Table 1). Mass spectrometric detection was conducted in ESI^−^ mode over m/z 100–1,000. Key annotated peaks corresponding to the major bioactive constituents of EAEPOL are labeled with their retention times (tR, min) and accurate m/z values, including: ginkgetin (tR = 13.27 min, m/z 551.0814), myricitrin (tR = 5.13 min, m/z 463.0750), cyanin (tR = 6.09 min, m/z 447.0839), and amentoflavone (tR = 9.87 min, m/z 537.0654).

The chemical constituents of EAEPOL were comprehensively profiled using HPLC-Q-TOF-MS/MS operating in ESI-. A representative total ion chromatogram (TIC) of EAEPOL is presented in Figure 1, which displayed a complex chromatographic profile with numerous peaks eluting over a 40-min period, indicating the presence of a diverse repertoire of chemical components with varying polarities in the extract. The base peak intensity (BPI) chromatogram identified several prominent peaks, with the most intense signal detected at a retention time (tR) of 13.27 min (m/z 551.0814), which was unambiguously identified as ginkgetin, a characteristic biflavonoid marker compound of *Platycladus orientalis*.

Based on accurate mass measurements (mass error <10 ppm), MS/MS fragmentation pattern matching against public databases (e.g., MassBank, HMDB) and an in-house library, a total of 60 unique chemical compounds were tentatively annotated in EAEPOL. These compounds were classified into five major categories, including flavonoids, biflavonoids, phenolic acids, terpenoids, and lignans. A comprehensive list of all annotated compounds, including their chemical formulas, retention times, observed m/z values, relative abundances (response values), mass errors, and adduct types, is provided in the Supplementary Material (Supplementary Table S1).

Semi-quantitative analysis based on peak area integration (response values) demonstrated that flavonoids and biflavonoids were the predominant phytochemical classes in EAEPOL. The four most abundant compounds were identified as ginkgetin (tR = 13.27 min, response = 524,047), cyanin (tR = 6.09 min, response: 519,462), myricitrin (tR = 5.13 min, response = 486,265), and amentoflavone (tR = 9.87 min, response = 468,951).

### 3.2 EAEPOL reduces random blood glucose levels in a time-dependent manner

**FIGURE 2:**
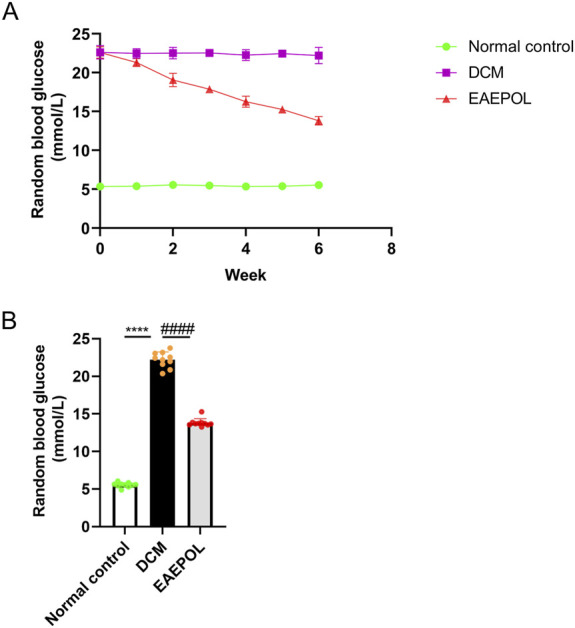
EAEPOL ameliorates random blood glucose in a time-dependent manner in STZ-induced diabetic cardiomyopathy (DCM) mice. **(A)** Longitudinal monitoring of random blood glucose levels in normal control, DCM model, and EAEPOL-treated mice over 6 weeks of intervention. Data are presented as mean ± SD (n = 10 per group). **(B)** Random blood glucose levels at the 6-week endpoint, with individual data points overlaid on the bar graph to show distribution. Data are presented as mean ± SD (n = 10 per group). Intergroup comparisons were performed by one-way ANOVA followed by Tukey’s HSD *post hoc* test. ****P < 0.0001 vs. normal control group; ####*P* < 0.0001 vs. DCM group.

To evaluate whether EAEPOL exerts metabolic benefits *in vivo*, random blood glucose levels were monitored weekly throughout the intervention. As depicted in Figure 2, mice in the DCM group exhibited sustained and marked hyperglycemia compared with the control group (*P* < 0.0001). EAEPOL treatment induced a progressive, time-dependent reduction in blood glucose relative to the DCM group, with significant differences observed as early as 1 week (*P* < 0.0001). At the end of the 6-week intervention, EAEPOL treatment resulted in a marked reduction in random blood glucose levels compared with the DCM group (*P* < 0.0001), although levels remained significantly higher than those in the normal control group (*P* < 0.0001), demonstrating a robust yet partial hypoglycemic effect. These data indicate that EAEPOL ameliorates hyperglycemia in diabetic mice, which may contribute to its cardioprotective effects in DCM.

### 3.3 EAEPOL attenuates ventricular remodeling and restores cardiac function in murine DCM

**FIGURE 3:**
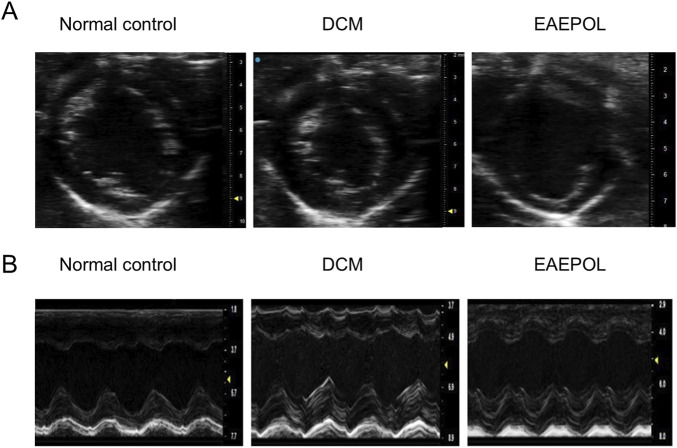
EAEPOL ameliorates cardiac structural and functional abnormalities in a mouse model of diabetic cardiomyopathy (DCM). **(A)** Representative short-axis M-mode echocardiographic images of the left ventricle in rats from the normal control group, DCM model group, and EAEPOL-treated group. These cross-sectional views illustrate the gross morphological changes in the left ventricular chamber. **(B)** Representative long-axis M-mode echocardiographic tracings of the left ventricle in the same three groups. These tracings are used for quantitative assessment of cardiac functional parameters, including left ventricular ejection fraction (LVEF) and ejection fraction (EF). Note: Echocardiography was performed to evaluate left ventricular structure and function in all experimental groups. The DCM group exhibited characteristic features of DCM, including left ventricular dilatation and impaired systolic function, which were significantly attenuated by EAEPOL treatment.

Ventricular hypertrophy and cardiac dysfunction are hallmark pathological features of DCM. To investigate whether EAEPOL ameliorates left ventricular (LV) remodeling and preserves cardiac function in DCM mice, key cardiac structural and functional parameters were quantified via B-mode and M-mode echocardiography. Compared with the normal control group, DCM mice exhibited significant increases in LVAWs and LVAWd, as well as LVPWs and LVPWd (all *P* < 0.05) (Table 3; Figure 3). Conversely, HR and EF were markedly reduced (*P* < 0.05 and *P* < 0.01, respectively) (Table 3). Critically, EAEPOL intervention effectively reversed these DCM-associated pathological alterations: Relative to the DCM group, EAEPOL-treated mice showed significant reductions in LVAWd, LVAWs, LVPWd, and LVPWs (all *P* < 0.05), while HR and EF were substantially improved (*P* < 0.01) (Table 3; Figure 3). These findings confirm that EAEPOL exerts a protective effect against DCM-induced ventricular remodeling and cardiac dysfunction, which is consistent with its potential as a cardioprotective agent.

**TABLE 3:** EAEPOL ameliorates cardiac dysfunction in DCM mice (Mean ± SD.*n* =10).

​	Normal control	DCM	EAEPOL
HR (bpm)	418.92 ± 23.03	322.79 ± 10.96^*^	415.3 ± 18.79^##^
EF (%)	72.19 ± 13.71	47.19 ± 12.35^**^	66.19 ± 7.40^##^
LVAWs (mm)	1.19 ± 0.10	1.49 ± 0.19^*^	1.20 ± 0.18^#^
LVAWd (mm)	0.84 ± 0.21	1.27 ± 0.27^*^	0.89 ± 0.27^#^
LVPWs (mm)	1.29 ± 0.12	1.39 ± 0.10^*^	1.16 ± 0.09^#^
LVPWd (mm)	0.74 ± 0.13	1.05 ± 0.17^*^	0.88 ± 0.10^#^

^*^ *P* < 0.05.

^**^ *P* < 0.01 vs*.* Normal control group.

^#^ *P* < 0.05.

^##^ *P* < 0.01 vs. DCM, group.

### 3.4 EAEPOL attenuates myocardial fibrosis in a murine DCM model

Myocardial fibrosis is a core pathological hallmark driving disease progression in DCM. To evaluate the potential anti-fibrotic effect of EAEPOL, we performed a histological and biochemical analysis of myocardial fibrosis in experimental mice. Myocardial tissue sections were subjected to H&E staining, Masson’s trichrome staining, Picro-Sirius red staining, and immunohistochemical staining for Collagen I and Collagen III to assess fibrotic pathological alterations. Concurrently, myocardial hydroxyproline content was quantified as a specific biochemical index of myocardial collagen deposition.

**FIGURE 4:**
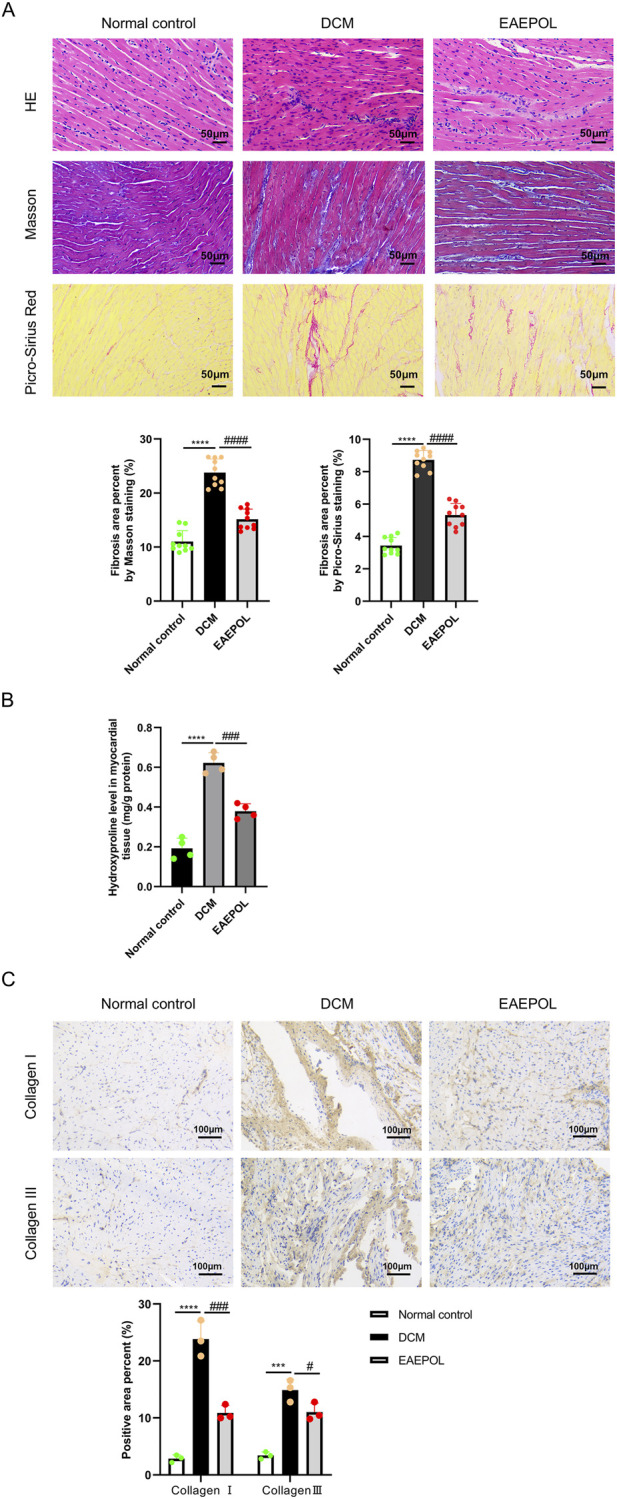
EAEPOL attenuates myocardial fibrosis in mice with diabetic cardiomyopathy (DCM). **(A)** Representative images of hematoxylin-eosin (HE), Masson’s trichrome, and Picro-Sirius red staining of myocardial paraffin sections from each group of mice. Scale bar = 50 μm (magnification, ×100). Top row: H&E staining showing proliferation of cardiac fibroblasts. Middle row: Masson’s trichrome staining highlighting interstitial collagen deposition (blue). Bottom row: Picro-Sirius red staining for collagen fibers (pink). The bar graphs quantify the fibrotic area percentage from Masson’s and Picrosirius red staining, respectively. **(B)** Quantitative analysis of hydroxyproline content in myocardial tissues (mg/g). **(C)** Representative immunohistochemical staining images and quantitative analysis of Collagen I and Collagen III in cardiac tissues. Data are presented as mean ± standard deviation (SD); n = 10 biologically independent mice for **(A)**, n = 4 biologically independent mice for **(B)**, and n = 3 biologically independent mice for **(C)**. Statistical significance was determined by one-way analysis of variance (ANOVA) followed by Tukey’s *post hoc* test. ***P < 0.001, ****P < 0.0001 vs. normal control group; #*P* < 0.05, ###*P* < 0.001, ####*P* < 0.0001 vs. DCM group.

The results demonstrated profound fibrotic remodeling in the myocardium of DCM mice compared with normal control mice. H&E staining demonstrated marked cardiac fibroblast proliferation in DCM mice (Figure 4A). A finding corroborated by Masson’s and Picro-Sirius Red staining, which showed extensive interstitial myocardial fibrosis and a significantly increased collagen volume fraction in the DCM group (*P* < 0.0001) (Figure 4A). Consistent with these morphological observations, myocardial hydroxyproline content was also significantly elevated in DCM mice (*P* < 0.0001) (Figure 4B). Furthermore, immunohistochemical analysis confirmed a substantial increase in the positive area ratio for both Collagen I and Collagen III (*P* < 0.001 or *P* < 0.0001) (Figure 4C).

Notably, EAEPOL treatment markedly attenuated all these DCM-induced fibrotic pathologies. In the EAEPOL-treated group, H&E staining indicated only minimal cardiac fibroblast proliferation, while Masson’s and Picro-Sirius Red staining revealed mild interstitial fibrosis accompanied by a significant reduction in the collagen-positive area ratio compared with the DCM group (*P* < 0.0001) (Figure 4A). In parallel, myocardial hydroxyproline content was significantly reduced following EAEPOL intervention (*P* < 0.001) (Figure 4B). Additionally, the immunohistochemical-positive areas for Collagen I and Collagen III were also significantly diminished in EAEPOL-treated mice (*P* < 0.001 and *P* < 0.05 respectively) (Figure 4C). These histological and biochemical data collectively confirm the potent anti-fibrotic activity of EAEPOL in the DCM myocardium, which may contribute to its cardioprotective effects.

### 3.5 EAEPOL preserves myocardial endothelial integrity and inhibits myofibroblast activation in murine DCM

Endothelial integrity is critical for maintaining normal myocardial structure and function, while myofibroblast activation is a key driver of myocardial fibrosis in DCM. To further elucidate the anti-fibrotic mechanism of EAEPOL, we analyzed the expression of endothelial integrity markers and myofibroblast activation markers in myocardial tissues. The protein expression levels of the endothelial cell marker CD31 and the myofibroblast activation marker α-SMA were analyzed by Western blot. The mRNA expression levels of the endothelial marker Cadherin-5 (CDH5) and the fibrosis-related marker Fibroblast-specific protein 1 (FSP1) were quantified by qRT-PCR. The spatial distribution and expression of α-SMA were further assessed by immunofluorescence staining.

**FIGURE 5:**
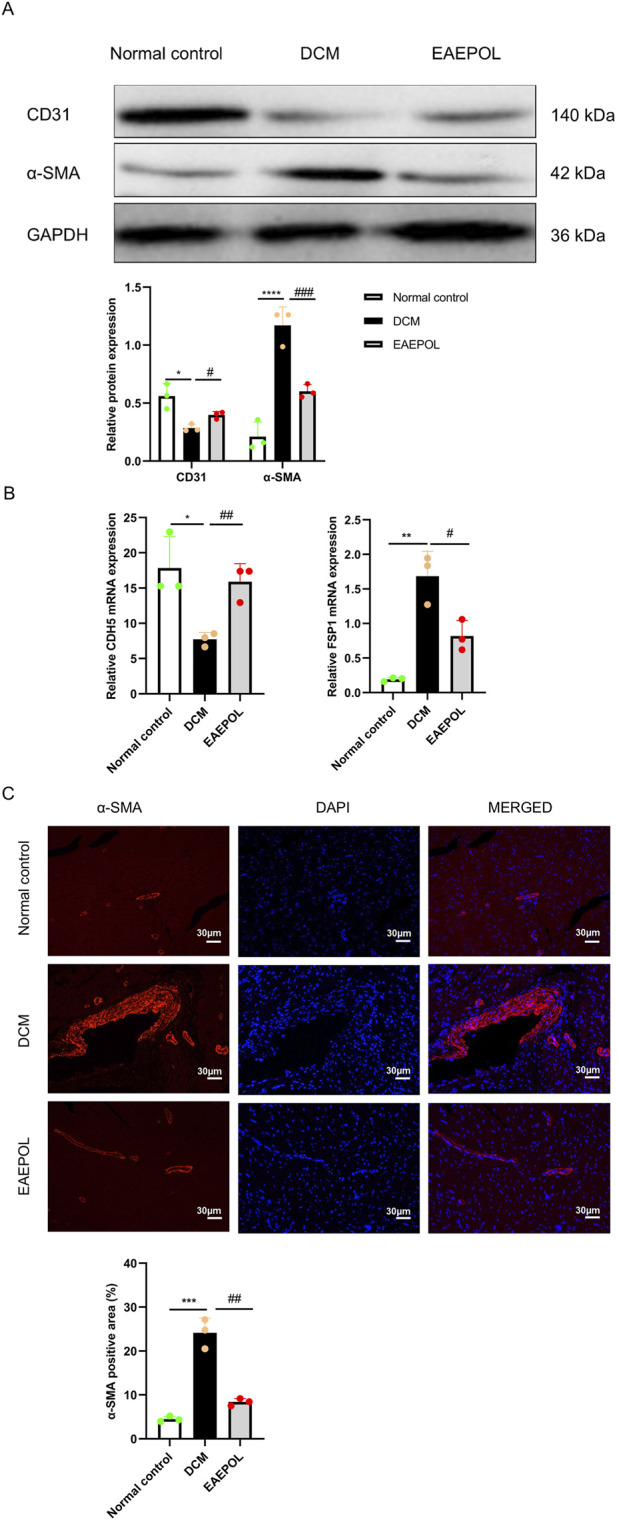
EAEPOL preserves endothelial integrity and inhibits myofibroblast activation in mice with diabetic cardiomyopathy (DCM). **(A)** Representative Western blot images (top panel) and quantitative analysis (bottom panel) of the endothelial integrity marker cluster of differentiation 31 (CD31) and the myofibroblast activation marker α-smooth muscle actin (α-SMA). **(B)** Quantitative reverse transcription polymerase chain reaction (qRT-PCR) analysis of the endothelial junction marker cadherin 5 (CDH5) and the fibrosis-related marker fibroblast-specific protein 1 (FSP1). **(C)** Representative immunofluorescence staining images of α-SMA (red) counterstained with 4′,6-diamidino-2-phenylindole (DAPI, blue) in cardiac tissue (scale bar = 30 μm). Data are presented as mean ± standard deviation (SD); n = 3 independent experiments. Statistical significance was determined by one-way analysis of variance (ANOVA) followed by Tukey’s *post hoc* test. *P < 0.05, **P < 0.01, ***P < 0.001, ****P < 0.0001 vs. normal control group; #*P* < 0.05, ##*P* < 0.01, ###*P* < 0.001 vs. DCM group.

Analysis of myocardial tissues from DCM mice revealed a significant downregulation of both the CD31 protein (*P* < 0.05) (Figure 5A) and CDH5 mRNA levels (*P* < 0.05) (Figure 5B), coupled with a marked upregulation of α-SMA protein (*P* < 0.0001) (Figure 5A) and FSP1 mRNA expression (*P* < 0.01) (Figure 5B), compared with the normal control group. This alteration in marker expression indicates impaired endothelial integrity and enhanced myofibroblast activation in DCM myocardium, which was further corroborated by immunofluorescence staining, which demonstrated a substantial increase in the α-SMA-positive area in the DCM group (*P* < 0.001) (Figure 5C).

EAEPOL treatment effectively reversed these DCM-induced pathological changes. Compared with the DCM group, EAEPOL administration significantly upregulated CD31 protein (*P* < 0.05) (Figure 5A) and CDH5 mRNA levels (*P* < 0.01) (Figure 5B), indicating preservation of myocardial endothelial integrity. Conversely, EAEPOL markedly downregulated the expression of α-SMA protein (*P* < 0.001) (Figure 5A) and FSP1 mRNA (*P* < 0.05) (Figure 5B), suggesting inhibition of myofibroblast activation and fibrosis progression. Consistently, immunofluorescence quantification demonstrated a marked reduction in the α-SMA-positive area following EAEPOL treatment (*P* < 0.01) (Figure 5C). These data collectively suggest that EAEPOL attenuates cardiac fibrosis in DCM at least in part by preserving endothelial integrity and inhibiting myofibroblast activation.

### 3.6 EAEPOL activates the Nrf2/HO-1 pathway to mitigate myocardial oxidative stress in a murine DCM model

Myocardial oxidative stress is a key upstream pathological driver of DCM, contributing to myocardial fibrosis and cardiac contractile dysfunction, to investigate the antioxidant potential of EAEPOL in DCM, we quantified T-AOC, SOD activity, and MDA content in murine myocardial tissues. Subsequently, to elucidate the underlying molecular mechanism of EAEPOL’s antioxidant effects, we examined the involvement of the Nrf2/HO-1 pathway.

**FIGURE 6:**
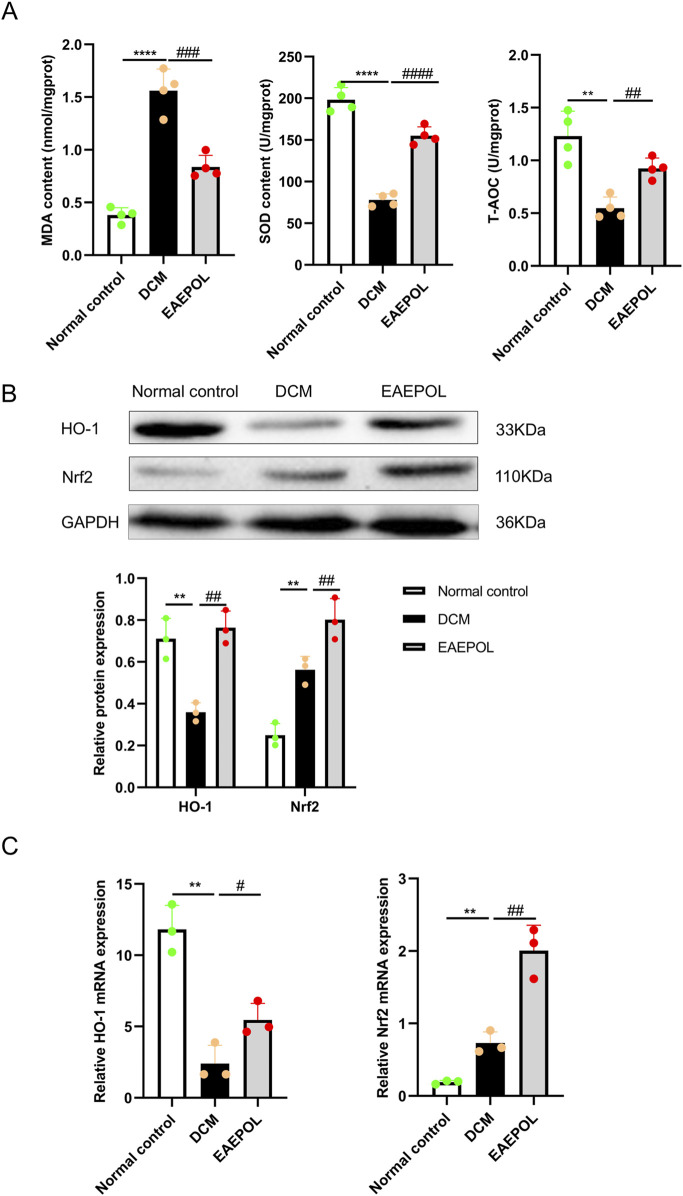
EAEPOL attenuates myocardial oxidative stress in mice with diabetic cardiomyopathy (DCM). **(A)** Myocardial levels of malondialdehyde (MDA), superoxide dismutase (SOD) activity, and total antioxidant capacity (T-AOC) in each indicated group (n = 4 per group). Data are presented as mean ± standard deviation (SD). **(B)** Representative Western blot images (top panel) and quantitative analysis (bottom panel) of heme oxygenase-1 (HO-1) and nuclear factor erythroid 2-related factor 2 (Nrf2) protein expression in mouse myocardium (n = 3 independent experiments). Data are presented as mean ± SD. **(C)** Quantitative reverse transcription polymerase chain reaction (qRT-PCR) analysis of HO-1 and Nrf2 mRNA expression. Data are presented as mean ± SD; n = 3 independent experiments. Statistical significance was determined by one-way analysis of variance (ANOVA) followed by Tukey’s *post hoc* test. **P < 0.01, ****P < 0.0001 vs. normal control group; #*P* < 0.05, ##*P* < 0.01, ###*P* < 0.001, ####*P* < 0.0001 vs. DCM group.

The DCM group exhibited a profound impairment of the myocardial antioxidant defense system compared with the normal control group, as evidenced by a significant increase in myocardial MDA levels (*P* < 0.0001), concomitant with marked reductions in both SOD activity and T-AOC (*P* < 0.0001, *P* < 0.01) (Figure 6A). EAEPOL treatment significantly attenuated these oxidative stress-related alterations, reducing MDA content (*P* < 0.001) and restoring SOD activity and T-AOC (*P* < 0.0001, *P* < 0.01) (Figure 6A), highlighting the potent *in vivo* antioxidant capacity of EAEPOL in the DCM myocardium.

To determine whether the antioxidant and cardioprotective effects of EAEPOL was mediated through the Nrf2/HO-1 signaling pathway, we analyzed the protein and mRNA levels of Nrf2 and HO-1 by Western blotting and qRT-PCR respectively. Myocardial tissues from DCM mice showed a significant downregulation of HO-1 and concomitant upregulation of Nrf2 expression at both the protein and mRNA levels compared with the normal control group (*P* < 0.01 for both) (Figures 6B,C). This dysregulation of the Nrf2/HO-1 pathway in DCM myocardium may contribute to impaired antioxidant defense and exacerbated oxidative stress. EAEPOL intervention robustly modulated the Nrf2/HO-1 pathway in the DCM myocardium, resulting in a significant upregulation of Nrf2 and HO-1 at the protein and mRNA levels (*P* < 0.05, *P* < 0.01) (Figures 6B,C). Collectively, these results demonstrate that EAEPOL alleviates oxidative stress in DCM hearts, at least in part by activating the Nrf2/HO-1 antioxidant signaling pathway.

## 4 Discussion

### 4.1 Phytochemical profiling of EAEPOL and its cardioprotective efficacy in a type 1 DCM model

LC-MS/MS analysis comprehensively profiled the phytochemical composition of EAEPOL revealing a rich profile dominated by flavonoids, terpenoids, and phenolic acids. This phytochemical profile is consistent with the well-characterized metabolic signature of *Platycladus orientalis* (Shan et al., 2014b). Semi-quantitative analysis identified ginkgetin, myricitrin, and amentoflavone as the three most abundant constituents in EAEPOL, this finding is consistent with previous reports showing that these biflavonoids and flavonol glycosides serve as the characteristic and major bioactive components of this species (Shan et al., 2014b; Lu et al., 2006); notably, amentoflavone and myricitrin are widely recognized as chemical markers for the quality control of *Platycladus orientalis* leaf extracts (Shan et al., 2014b).

Of particular interest was the detection of cyanin at a remarkably high relative abundance (response value: 519,462). While anthocyanins are ubiquitously distributed in the plant kingdom, they have not previously been recognized as predominant components in *Platycladus orientalis* leaf extracts. The elevated cyanin content in our preparation is likely attributable to the use of fresh plant material: flavonoid accumulation, including anthocyanins, in *Platycladus orientalis* leaves exhibits pronounced seasonal and physiological variability (Sęczyk et al., 2022). Fresh leaves inherently retain labile metabolites such as anthocyanins, which are susceptible to degradation during prolonged storage or drying procedures. Additionally, ethyl acetate, a semi-polar solvent, is known to efficiently enrich phenolic and flavonoid compounds (Hao et al., 2023), a property that may have further contributed to the high cyanin levels observed herein.

The well-established STZ-induced murine model of type 1 DCM recapitulated key pathological features of human disease (Furman, 2021), providing a clinically relevant platform to evaluate the therapeutic efficacy of EAEPOL. The observed cardioprotective effects, including lowered blood glucose, improved cardiac function, attenuated ventricular hypertrophy, and reduced myocardial fibrosis, establish EAEPOL as a promising therapeutic candidate and provide a phenotypic anchor for subsequent mechanistic dissection.

Ginkgetin, myricitrin, and amentoflavone—the three most abundant flavonoids in EAEPOL—have all been implicated in the regulation of core pathological processes underlying DCM, including oxidative stress, inflammation, fibrosis, and metabolic jdysregulation (Li et al., 2025; Liu et al., 2020; Zhang et al., 2017; Yu et al., 2017; Qin et al., 2018). While ginkgetin has been primarily studied in the context of myocardial infarction (Li et al., 2025), and myricitrin directly in DCM models (Zhang et al., 2017), the collective evidence points to a common mechanism involving suppression of oxidative stress and fibrosis. This convergence suggests that the multi-flavonoid composition of EAEPOL may target complementary nodes within the same pathological network, with the potential for synergistic therapeutic effects—a hypothesis that warrants direct experimental testing.

The high cyanin abundance in EAEPOL is also of particular interest, given the accumulating evidence supporting its cardioprotective potential. Cyanin protects against endotoxin-induced myocardial injury by preserving cardiac function, reducing cardiomyocyte death, and modulating mitochondrial dynamics (Li et al., 2018), and a comprehensive review by Liobikas *et al.* (Liobikas et al., 2016) further highlighted the broad cardioprotective effects of anthocyanins, including cyanidin.

Despite these promising findings, several critical questions remain unanswered. The present study did not quantify the plasma or myocardial tissue concentrations of ginkgetin, myricitrin, or amentoflavone following EAEPOL administration, nor did it establish a direct correlation between their tissue levels and the observed pharmacological effects. Future research should focus on characterizing the pharmacokinetic profiles and tissue distribution of these key compounds, which would provide critical insights into their *in vivo* fate and inform rational strategies to enhance their bioavailability. Additionally, comprehensive evaluation of the toxicity and safety profiles of EAEPOL remains to be conducted. Future studies should perform systematic assessments of the safety, potential side effects, and long-term biosafety of EAEPOL, incorporate sex-stratified analyses, larger sample sizes, and extended follow-up data, and further validate its translational potential and clinical applicability as a candidate therapeutic agent.

### 4.2 EAEPOL alleviates myocardial oxidative stress in type 1 DCM via activating the Nrf2/HO-1 signaling pathway

Myocardial oxidative stress, driven by excessive ROS generation, is a pivotal pathogenic mediator of DCM. Under hyperglycemic conditions, ROS are predominantly produced via NADPH oxidases and the mitochondrial electron transport chain (Ye et al., 2025); when cellular antioxidant defense mechanisms are overwhelmed, ROS induce oxidative damage to lipids, proteins, and DNA, thereby exacerbating myocardial injury. Consistent with this pathological paradigm, our data confirmed robust myocardial oxidative stress in DCM mice, which likely represents a key mechanistic driver of the observed cardiac dysfunction and fibrosis. EAEPOL treatment effectively reversed this oxidative imbalance, demonstrating its potent *in vivo* antioxidant activity in the DCM microenvironment.

To elucidate the underlying molecular mechanism, we focused on the Nrf2/HO-1 signaling pathway—a master regulator of the cellular adaptive antioxidant response. Under basal conditions, Nrf2 is sequestered in the cytoplasm by its endogenous repressor Kelch-like ECH-associated protein 1 (Keap1). Following oxidative challenge, Nrf2 dissociates from Keap1, translocates to the nucleus, and transactivates downstream cytoprotective genes including heme oxygenase-1 (HO-1), thereby exerting antioxidant, anti-inflammatory, and anti-apoptotic effects (Calvert et al., 2009). Notably, our study identified a dysregulated Nrf2/HO-1 pathway in the myocardium of DCM mice, characterized by elevated Nrf2 expression yet diminished HO-1 levels. This phenomenon, termed Nrf2 signaling uncoupling, has been previously documented in the diabetic heart (Chapple et al., 2016) and is mechanistically attributed to nuclear accumulation of Bach1—a transcriptional repressor that competes with Nrf2 for binding to antioxidant response element (ARE) sites on the HO-1 promoter, thus abrogating HO-1 transcription despite Nrf2 activation (Chapple et al., 2016). This impaired antioxidant response likely exacerbates myocardial oxidative stress and injury in the DCM model. EAEPOL treatment significantly upregulated myocardial protein expression of both Nrf2 and HO-1, suggesting that EAEPOL modulates the Nrf2/HO-1 signaling pathway to promote HO-1 induction in the DCM microenvironment, thereby activating this endogenous antioxidant defense system to counteract oxidative damage. Collectively, these findings indicate that EAEPOL attenuates myocardial oxidative stress—and consequently ameliorates cardiac dysfunction and fibrosis—at least partially via activation of the Nrf2/HO-1 signaling pathway.

These results align with prior investigations into the antioxidant properties of *Platycladus orientalis* leaf extracts: Gu et al. identified EAEPOL as the most potent antioxidant fraction relative to petroleum ether and n-butanol extracts of this plant (Gu and Sun, 2015). LC-MS/MS profiling has further revealed that EAEPOL is enriched in flavonoids (over thirty distinct flavonoid compounds), all of which possess well-characterized antioxidant activity. Importantly, the principal bioactive constituents of EAEPOL—ginkgetin, myricitrin, and amentoflavone—as well as the unexpectedly highly abundant cyanin, have each been reported to activate the Nrf2/HO-1 pathway and exert robust antioxidant effects (Li et al., 2025; Zhang et al., 2017; Yu et al., 2017; Li et al., 2018). The coexistence of multiple Nrf2-activating flavonoids within EAEPOL likely underpins the potent *in vivo* antioxidant efficacy observed in our DCM model. In addition, EAEPOL significantly reduced random blood glucose levels in a time-dependent manner. As a key upstream risk factor for diabetic cardiomyopathy, sustained hyperglycemia aggravates myocardial oxidative stress and fibrosis. By alleviating chronic hyperglycemia, EAEPOL reduced myocardial metabolic burden and may partially contributed to its cardioprotective effect.

Notwithstanding these promising findings, the present study has several notable limitations. While our data demonstrate that EAEPOL enhances myocardial antioxidant defenses and modulates Nrf2/HO-1 signaling, definitive evidence that the cardioprotective effects of EAEPOL are directly mediated via this pathway would require investigations in Nrf2 knockout mice. Furthermore, the absence of a direct comparison between EAEPOL and established pharmacological Nrf2 activators limits the interpretation of its relative efficacy in activating Nrf2 signaling. Such comparative evaluations represent an important direction for future pharmacological studies. Additionally, systematic evaluations of the individual *in vivo* antioxidant efficacy of ginkgetin, myricitrin, and amentoflavone—along with delineation of their specific contributions to Nrf2/HO-1 pathway activation—are warranted to clarify whether the therapeutic effects of EAEPOL stem from additive or synergistic interactions among these bioactive flavonoid constituents.

### 4.3 EAEPOL alleviates diabetic myocardial fibrosis by preserving endothelial integrity and inhibiting myofibroblast activation

Myocardial fibrosis is a core pathological feature of DCM, driven by excessive collagen deposition and myofibroblast activation, while endothelial integrity is critical for maintaining normal myocardial microcirculation and preventing fibrotic remodeling. Endothelial dysfunction and myofibroblast activation are closely interconnected in DCM: impaired endothelial integrity disrupts myocardial blood supply, promotes inflammatory cell infiltration, and triggers myofibroblast activation, thereby accelerating collagen accumulation and interstitial fibrosis (Huang et al., 2025; Feng et al., 2024; Gaikwad et al., 2020; Wang et al., 2023). Our findings confirm impaired myocardial endothelial integrity and enhanced myofibroblast activation in DCM mice, as evidenced by reciprocal alterations in endothelial integrity markers (CD31, CDH5) and myofibroblast activation markers (α-SMA, FSP1). Notably, EAEPOL treatment effectively reversed these pathological changes, indicating that preservation of endothelial integrity and inhibition of myofibroblast activation contribute to the anti-fibrotic and cardioprotective effects of EAEPOL. These observations identify EAEPOL as a potential modulator of two interconnected pathological processes that drive DCM-associated myocardial fibrosis. In line with current methodological guidelines for rigorous evaluation of cardiac fibrosis (Piera-Velazquez and Jimenez, 2019; Kovacic et al., 2019), our integrated transcriptional and translational analysis—combining Western blot, qRT-PCR, and immunofluorescence—provides compelling evidence that EAEPOL mitigates fibrotic remodeling by targeting these key processes.

Oxidative stress is increasingly recognized as a key upstream trigger of both endothelial dysfunction and myofibroblast activation (Piera-Velazquez and Jimenez, 2019). Accumulating evidence indicates that non-coding RNAs (miRNAs and lncRNAs) participate in the post-transcriptional regulation of these processes, and oxidative stress can directly modulate the expression of these regulatory RNAs (Giordo et al., 2021). Giordo et al. demonstrated that ROS activate TGF-β-mediated fibrotic signaling to promote tissue fibrosis (Giordo et al., 2021), while Deng et al. reported that oxidative stress induces endothelial dysfunction in human umbilical vein endothelial cells, leading to cellular injury and pro-fibrotic responses (Deng et al., 2022). These collective findings substantiate a causal link between oxidative stress, endothelial dysfunction, and myofibroblast activation. In our DCM model, myocardial oxidative stress, endothelial impairment, and myofibroblast activation were all markedly upregulated, implicating hyperglycemia-induced oxidative injury as a critical upstream mediator of cardiac fibrosis via dysregulation of these interconnected processes.

Despite these informative findings, the present study has several methodological limitations that merit acknowledgment. First, immunofluorescence co-localization of endothelial markers (e.g., CD31) with myofibroblast markers (e.g., α-SMA) would provide more direct evidence of the spatial relationship between endothelial dysfunction and myofibroblast activation in the DCM myocardium. Second, myofibroblast activation and endothelial function are dynamic processes governed by intricate signaling networks and key transcriptional regulators (e.g., Snail, Slug, Twist for myofibroblast activation; eNOS, VEGF for endothelial function). Future investigations should characterize the expression of these regulators and interrogate the involvement of canonical upstream signaling pathways that drive both endothelial dysfunction and myofibroblast activation in DCM. Third, while the literature extensively documents oxidative stress as a key trigger of endothelial dysfunction and myofibroblast activation, the present study lacks direct experimental evidence validating this mechanistic link in the context of DCM; for example, whether ROS scavenger treatment can reverse endothelial impairment and myofibroblast activation in the diabetic myocardium remains to be elucidated.

### 4.4 Future perspectives and translational implications

The present study demonstrates that EAEPOL ameliorates type 1 DCM by inhibiting two interconnected pathological processes—oxidative stress and fibrotic remodeling (via preservation of endothelial integrity and inhibition of myofibroblast activation)—both of which are well-documented key mediators of cancer therapy-induced cardiotoxicity (L'Abbate et al., 2020; Slavcheva and Angelov, 2023). Excessive ROS production, a pathological hallmark shared by DCM and cancer therapy-related cardiac injury, drives cardiomyocyte damage and aberrant fibrotic remodeling (L'Abbate et al., 2020; Hara, 2025). The antioxidant capacity of EAEPOL, mediated in part through activation of the Nrf2/HO-1 pathway, directly targets this common pathological driver, consistent with prior studies linking oxidative stress to anthracycline-induced cardiomyopathy (L'Abbate et al., 2020). Notably, anthracycline-based chemotherapy, widely used in various cancers, is limited by dose-dependent cardiotoxicity that shares pathological features with DCM (Kuang et al., 2024). Given the central role of oxidative stress in its pathogenesis, the Nrf2/HO-1-mediated antioxidant effects of EAEPOL, initially characterized in DCM, may represent a translatable strategy for mitigating anthracycline-induced cardiomyopathy (Yang et al., 2025).

Beyond oxidative stress, endothelial dysfunction and myofibroblast activation represent additional convergent mechanisms linking DCM and cancer therapy-induced cardiotoxicity. In the present study, EAEPOL preserved myocardial endothelial integrity and inhibited myofibroblast activation in DCM, as evidenced by the restoration of endothelial marker expression and suppression of myofibroblast activation marker upregulation. Endothelial dysfunction and excessive myofibroblast activation are also critical processes in immune checkpoint inhibitor (ICI)-associated myocarditis and HER2-targeted therapy-induced cardiac fibrosis (Slavcheva and Angelov, 2023), thus establishing a mechanistic rationale for extending the protective effects of EAEPOL—observed in DCM—to cancer therapy-related cardiotoxicity. These mechanistic convergences align with emerging evidence underscoring overlapping pathophysiological pathways between metabolic heart disease, such as DCM, and cancer therapy-related cardiac injury (Hara, 2025). Collectively, the ability of EAEPOL to target both oxidative stress (via Nrf2/HO-1 modulation) and fibrotic remodeling (via preservation of endothelial integrity and inhibition of myofibroblast activation)—two shared pathogenic drivers between DCM and cancer therapy-induced cardiac injury—aligns with the overlapping pathophysiological mechanisms of these conditions.

In conclusion, EAEPOL holds distinct therapeutic advantages for the management of DCM, attributed to its multiple beneficial activities, favorable preclinical safety profile, and unique flavonoid-rich composition. Nevertheless, its clinical translation is hindered by several critical limitations, including the absence of clinical evidence, inherent compositional complexity, and poorly defined *in vivo* bioavailability of its active constituents. Furthermore, direct comparative efficacy studies between EAEPOL and first-line DCM therapeutics (e.g., metformin, SGLT2 inhibitors) have not yet been performed. While recent research has demonstrated that seabuckthorn extract exerts synergistic cardioprotective effects with metformin in DCM models, the potential pharmacodynamic interactions and comparative therapeutic efficacy of EAEPOL in combination with or as an alternative to conventional pharmacotherapies remain unexplored. Future investigations should therefore prioritize four key directions: (i) comprehensive *in vivo* pharmacokinetic and pharmacodynamic profiling of the principal bioactive constituents of EAEPOL to clarify their tissue distribution and mechanism-of-action contributions; (ii) rigorous comparative and combinatorial efficacy studies with standard-of-care DCM therapeutics to define optimal clinical application strategies; (iii) translational advancement of preclinical findings to early-phase clinical trials to evaluate safety and preliminary efficacy in human subjects; and (iv) inclusion of multiple time-point analyses to distinguish whether EAEPOL acts as a preventive intervention or a therapeutic reversal agent in DCM; (v) in-depth research on the therapeutic effects and underlying mechanisms of EAEPOL in type 2 diabetes mellitus (T2DM)-associated DCM, so as to improve its clinical adaptability and expand its application scope in the comorbidity of T2DM and DCM. Given the substantial overlap in pathophysiological mechanisms with cancer therapy-induced cardiotoxicity, these findings offer novel insights into the potential application of EAEPOL in contexts requiring cardioprotection during cancer treatment, particularly for anthracycline-induced cardiomyopathy and ICI-associated myocarditis.

## Data Availability

The original contributions presented in the study are included in the article/Supplementary Material, further inquiries can be directed to the corresponding author.
