# Supplementary material for: Ethyl acetate extract from Platycladus orientalis leaves ameliorates diabetic cardiomyopathy via alleviating oxidative stress and suppressing myocardial fibrosis
Source: Front Pharmacol. 2026 May 13;17:1771512. doi: 10.3389/fphar.2026.1771512 (PMC13212482; doi:10.3389/fphar.2026.1771512)
Supplement: Supplementary file 1 [file Table1.docx]

**Table S1. Unique Compounds Identified in the Ethyl Acetate Extract of Platycladus orientalis Leaves by LC-MS/MS**

| **No.** | **Compound Name** | **Formula** | **tR (min)** | **Observed m/z** | **Response** | **Mass Error (ppm)** | | **Adduct** |
| --- | --- | --- | --- | --- | --- | --- | --- | --- |
| 1 | Fargesin flavonol glycoside A | C30H26O13 | 1.06 | 593.129 | 28,063 | -1.8 | | [M-H] ⁻ |
| 2 | Procyanidin B3 | C30H26O12 | 2.32 | 577.1346 | 220,579 | -1 | | [M-H] ⁻ |
| 3 | Fraxin | C16H18O10 | 2.37 | 369.0847 | 9,265 | 5.5 | | [M-H] ⁻ |
| 4 | Safflomin C | C30H30O14 | 2.38 | 613.1538 | 11,308 | -4.1 | | [M-H] ⁻ |
| 5 | 3-O-trans-p-Coumaroylquinic acid | C16H18O8 | 2.53 | 337.0962 | 53,167 | 9.7 | | [M-H] ⁻ |
| 6 | Gentianoside A | C17H24O11 | 3.07 | 403.1266 | 42,661 | 5.1 | | [M-H] ⁻ |
| 7 | Swertiamarin | C17H24O9 | 3.57 | 371.1373 | 7,349 | 6.8 | [M-H] ⁻ | |
| 8 | 6-Acetylshanzhiside methyl ester | C19H28O12 | 3.66 | 447.1516 | 6,794 | 1.7 | [M-H] ⁻ | |
| 9 | Neoaesculin | C21H22O11 | 5.51 | 449.11 | 54,658 | 2.4 | [M-H] ⁻ | |
| 10 | Taxifolin-3'-O-β-D-glucopyranoside | C21H22O12 | 4.43 | 465.1054 | 142,198 | 3.3 | [M-H] ⁻ | |
| 11 | Cannabiscoumarin | C21H20O13 | 4.53 | 479.0837 | 14,212 | 1.3 | [M-H] ⁻ | |
| 12 | Ligustroside A | C25H34O12 | 4.53 | 525.1973 | 15,582 | -0.8 | [M-H] ⁻ | |
| 13 | Myricitrin | C21H20O12 | 5.13 | 463.0750 | 486,265 | 3.9 | [M-H] ⁻ | |
| 14 | Azadirachtin | C20H18O12 | 5.19 | 449.0737 | 5,436 | 2.6 | [M-H] ⁻ | |
| 15 | 2-Methoxy-4-acetylphenol 1-O-α-L-rhamnopyranosyl-(1''→6')-β-D-glucopyranoside | C21H30O12 | 5.43 | 473.1665 | 6,624 | 0.1 | [M-H] ⁻ | |
| 16 | Darendoside A | C19H28O11 | 5.62 | 431.1573 | 8,943 | 3.2 | [M-H] ⁻ | |
| 17 | 1-(4-Hydroxybenzyl)-4-methoxy-2,7-dihydroxyphenanthrene | C22H18O4 | 5.75 | 345.1121 | 9,545 | -3.4 | [M-H] ⁻ | |
| 18 | (-)-Secoisolariciresinol-4-O-β-D-glucoside | C26H36O11 | 5.84 | 523.2175 | 6,893 | -1.8 | [M-H] ⁻ | |
| 19 | (-)-Olivil-4''-O-β-D-glucopyranoside | C26H34O12 | 5.88 | 537.1971 | 15,500 | -1.2 | [M-H] ⁻ | |
| 20 | Cyanin | C21H20O11 | 6.09 | 447.0839 | 519,462 | 4.8 | [M-H] ⁻ | |
| **No.** | **Compound Name** | **Formula** | **tR (min)** | **Observed m/z** | **Response** | **Mass Error (ppm)** | | **Adduct** |
| 21 | Centaureidin | C19H24Cl2O7 | 6.17 | 433.0812 | 21,874 | -3.2 | | [M-H] ⁻ |
| 22 | Nepitrin | C22H22O12 | 6.26 | 477.1044 | 8,587 | 1.1 | | [M-H] ⁻ |
| 23 | Naringenin-4′-O-glucopyranoside | C21H22O10 | 6.29 | 433.115 | 35,079 | 2.3 | | [M-H] ⁻ |
| 24 | Ellagic acid-4-O-β-D-glucopyranoside | C23H22O13 | 6.3 | 505.0988 | 7,508 | 0 | | [M-H] ⁻ |
| 25 | Cimicifugic acid E | C21H20O10 | 6.91 | 431.1001 | 82,222 | 4 | | [M-H] ⁻ |
| 26 | Taraxasteryl glucoside | C21H34O9 | 7.23 | 429.2148 | 25,250 | 4.1 | | [M-H] ⁻ |
| 27 | Chrysoeriol-7-O-β-D-glucoside | C22H22O11 | 7.11 | 461.1096 | 5,887 | 1.4 | | [M-H] ⁻ |
| 28 | 1,3-Dihydroxy-2-hydroxymethylanthraquinone-3-O-β-D-xylopyranosyl-(1→6)-β-D-glucopyranoside | C26H28O14 | 6.81 | 563.1395 | 6,880 | -2 | [M-H] ⁻ | |
| 29 | Matairesinoside | C26H32O11 | 6.81 | 519.1869 | 12,223 | -0.5 | [M-H] ⁻ | |
| 30 | Cimicifugic acid C | C20H18O10 | 7.01 | 417.0845 | 39,372 | 4.3 | [M-H] ⁻ | |
| 31 | Bruceine A | C26H34O11 | 8.18 | 521.2021 | 7,233 | -1.5 | [M-H] ⁻ | |
| 32 | Schisantherin C | C30H36O10 | 7.5 | 555.2229 | 12,183 | -1.3 | [M-H] ⁻ | |
| 33 | Myricetin 3′,5′-dimethyl ether 3-O-β-D-glucopyranoside | C23H24O13 | 7.62 | 507.1147 | 20,192 | 0.5 | [M-H] ⁻ | |
| 34 | Mulberroside hydroperoxide | C25H24O8 | 7.73 | 451.1409 | 5,737 | 2.3 | [M-H] ⁻ | |
| 35 | Pterodontoside A | C21H32O8 | 8.68 | 411.2039 | 10,931 | 3.5 | [M-H] ⁻ | |
| 36 | 14-Deoxyandrographolide-19-β-glucoside | C26H40O9 | 9.47 | 495.2599 | 31,972 | -0.2 | [M-H] ⁻ | |
| 37 | Amentoflavone | C30H18O10 | 9.87 | 537.0654 | 468,951 | 1.4 | [M-H] ⁻ | |
| 38 | Sanleng acid | C18H34O5 | 10.9 | 329.2363 | 6,567 | 8.9 | [M-H] ⁻ | |
| 39 | Ephedradine B | C30H20O10 | 11.4 | 539.0983 | 64,048 | -0.2 | [M-H] ⁻ | |
| 40 | Pd-C-Ⅳ | C21H22O7 | 11.8 | 385.1314 | 6,583 | 5.6 | [M-H] ⁻ | |
| 41 | Ginkgetin | C31H20O10 | 13.27 | 551.0814 | 524,047 | 0.6 | [M-H] ⁻ | |
| 42 | Andrographoside | C26H40O8 | 12.8 | 479.2657 | 11,233 | 1.4 | [M-H] ⁻ | |
| **No.** | **Compound Name** | **Formula** | **tR (min)** | **Observed m/z** | **Response** | **Mass Error (ppm)** | | **Adduct** |
| 43 | Dihydroxyisopsoralen | C20H16O7 | 12.9 | 367.085 | 18,796 | 7.3 | | [M-H] ⁻ |
| 44 | 14-Deoxyandrographolide | C20H30O4 | 13.8 | 333.21 | 7,585 | 8.6 | | [M-H] ⁻ |
| 45 | Cryptomerin B | C32H22O10 | 15.1 | 565.1143 | 278,936 | 0.6 | | [M-H] ⁻ |
| 46 | 7β-(3-Ethyl-cis-crotonoyloxy)-14-hydroxynotonipetranone | C21H32O4 | 17.8 | 347.2262 | 137,913 | 9.9 | | [M-H] ⁻ |
| 47 | Ganoderic acid H | C32H44O9 | 18.7 | 571.2892 | 330,665 | -3.7 | | [M-H] ⁻ |
| 48 | Isotetrandrine | C38H42N2O6 | 18.5 | 621.3025 | 15,460 | 8.8 | | [M-H] ⁻ |
| 49 | Isosamarcandin | C29H38O6 | 18.8 | 481.2583 | 40,126 | -2.6 | | [M-H] ⁻ |
| 50 | Datiscetin H | C34H46O9 | 19 | 597.3031 | 18,627 | -6.4 | | [M-H] ⁻ |
| 51 | Periplogenin | C23H34O5 | 19.5 | 389.2354 | 9,472 | 5.4 | | [M-H] ⁻ |
| 52 | Atractylenolide dimer | C30H38O4 | 21.7 | 461.268 | 20,341 | -3.8 | | [M-H] ⁻ |
| 53 | Stemonaenone | C22H29NO5 | 22.8 | 386.1966 | 8,706 | -1.8 | | [M-H] ⁻ |
| 54 | Benzyl alcohol xylopyranosyl-(1→6)-glucopyranoside | C20H28O11 | 22.8 | 401.144 | 8,791 | -3.3 | | [M-H] ⁻ |
| 55 | Stigmasterol-3-O-β-D-glucoside | C35H58O6 | 27.8 | 573.4159 | 42,724 | -0.2 | | [M-H] ⁻ |
| 56 | 20(R)-Ginsenoside Rh2 | C36H62O8 | 29 | 621.4348 | 15,632 | -3.9 | | [M-H] ⁻ |
| 57 | Soybean cerebroside I_1 | C42H81NO9 | 30.3 | 712.5326 | 17,158 | -6.1 | | [M-H] ⁻ |
| 58 | Melia triol | C30H50O4 | 38.8 | 489.3615 | 5,715 | 6.1 | [M-H] ⁻ | |
| 59 | Picrasidine S | C30H28N4O4 | 8.87 | 507.2084 | 16,193 | 9.1 | [M-H] ⁻ | |
| 60 | 7β-(3-Ethyl-cis-crotonoyloxy)-14-hydroxynotonipetranone | C21H32O4 | 17.8 | 347.2262 | 137,913 | 9.9 | [M-H] ⁻ | |

Note: A total of 60 unique compounds were tentatively identified in the ethyl acetate extract of Platycladus orientalis leaves using liquid chromatography-tandem mass spectrometry (LC-MS/MS) in negative electrospray ionization mode (ESI-). The raw data contained 104 entries, which were de-duplicated by retaining only the entry with the highest response value (relative peak area) for each unique compound name. Identification was based on accurate mass measurement (mass error < 10 ppm) and comparison of MS/MS fragmentation patterns with data from public databases (MassBank, HMDB) and an in-house library. The observed ions were predominantly [M-H] ⁻ adducts. Compounds are listed in order of their retention time (tR).
